# Durability Evaluation of Outdoor Scrimbers Fabricated from Superheated Steam-Treated Bamboo Fibrous Mats

**DOI:** 10.3390/polym15010214

**Published:** 2022-12-31

**Authors:** Li Qin, Jinguang Wei, Minzhen Bao, Yanglun Yu, Wenji Yu

**Affiliations:** 1 Industry Development and Planning Institute, National Forestry and Grassland Administration, Beijing 100010, China; 2 Key Laboratory of State Forest and Grassland Administration on Wood Quality Improvement & High Efficient Utilization, School of Forestry and Landscape Architecture, Anhui Agricultural University, Hefei 230036, China; 3 Key Laboratory of High Efficient Processing of Bamboo of Zhejiang Province, China National Bamboo Research Center, Hangzhou 310012, China; 4 Scrimber Engineering and Technology Research Center of State Forestry and Grassland Administration, Research Institute of Wood Industry, Chinese Academy of Forestry, Beijing 100091, China

**Keywords:** superheated steam, bamboo scrimber, mechanical properties, dimensional stability, aging resistance, decay resistance, anti-mildew property

## Abstract

With natural texture and high performance, bamboo scrimber is one of the artificial lignocellulosic composites widely used in construction, furniture and other structural applications. However, it is vulnerable to the actions of water, ultraviolet radiation and fungus, which affect its durability, especially in the open. Here, bamboo was treated with superheated steam in an attempt to improve the durability of bamboo scrimbers. The chemical composition, mechanical properties, dimensional stability, aging resistance, decay resistance and anti-mildew properties were investigated at different temperatures (160~200 °C). After superheated steam treatment, the relative contents of holocellulose and α-cellulose in bamboo decreased. The bending strength and short-beam shearing strength slightly decreased as the temperature was raised while the modulus was essentially retained. The aging resistance in terms of thickness swelling rate (≤9.38%) was substantially improved. The decay resistance reached to the level of Grade I and can be dramatically enhanced by elevating temperature. The anti-mildew properties were also improved. To take together, superheated steam treatment remarkably improves the resistance of bamboo scrimbers to water, ultraviolet radiation, rot fungi and mildew with some concomitant reduction in mechanical properties. The results will permit outdoor construction using bamboo scrimbers more resistant to environmental damage.

## 1. Introduction

With great concern that climate change is threatening the development and survival of human beings, carbon-negative materials are being developed to nullify embodied CO_2_ emissions, in the meantime transforming constructions into net carbon storage structures on the basis of the life cycle [1]. Among them, bamboo scrimber is the most attractive one for structural applications [2]. Bamboo scrimber is produced by laminating the mechanically rolled bamboo (called bamboo fibrous mat) into a dense block in the presence of phenolic resin [3]. The natural characteristics and orientation of bamboo fibers remain during production. Thus, bamboo scrimber possesses a beautiful texture and excellent mechanical properties. It also possesses good dimensional stability because of the high hydrophobicity of cured phenolic resin [4]. In addition to the attractive features such as fast growth, high abundance and renewability of bamboo, bamboo scrimber is regarded as a promising carbon-negative structural material and thus has been extensively used in construction, furniture and other structural applications [5].

However, there are some liabilities, such as discoloration, durability and dimensional stability, which must be considered when the material is applied for outdoor construction. Bamboo consists of cell walls of fiber cells, parenchyma cells and vessel cells, whose lumens form an opened-pore alveolate structure [6]. The structure permits air and moisture transfer and microbe reproduction within bamboo. Cell walls are mainly composed of cellulose, hemicellulose and lignin, in which there are a large number of free hydroxyl and carboxyl groups endowing bamboo with strong moisture absorbability [7]. Bamboo contains abundant nutrients such as starch, protein, sugar and fat, which makes bamboo vulnerable to attack by water and microbes [8,9,10]. When bamboo is processed into a scrimber, degradation is inhibited due to the high density of the scrimber and the cladding effect of cured resin [11]. However, when bamboo scrimbers are exposed to external conditions, phenomena such as deformation, cracking, decay, mold and discoloration take place [12]. Therefore, the durability of bamboo scrimber strongly limits outdoor applications.

An effective way to improve durability is to modify bamboo using thermal treatment. Heat treatment is a commercially viable technology, which changes the physical structure and/or chemical compositions of bamboo at elevated temperatures to achieve designed changes [13]. Heat treatments of bamboo using stream, air or inert gas, oil, and water have been reported [14,15,16,17]. Among these, the use of superheated steam has been widely adopted due to its high feasibility for implementation, low pollution and high efficiency [18,19,20]. Generally, superheated steam treatment is conducted in a hypoxic environment where the temperature ranges from 160 to 240 °C. If the temperature is below 160 °C, no obvious structural change is found. As the temperature exceeds 240 °C, cellulose pyrolyzes violently, resulting in a substantial reduction in mechanical properties. At the temperature between these extremes, exothermic reactions occur [21], in which parts of the cell wall components undergo pyrolysis. The result is a remarkable enhancement in dimensional stability and microbe resistance, but a slight decrease in mechanical properties [22]. It has been demonstrated that bamboo hygroscopicity decreases as a result of the degradation of hygroscopic hemicellulose, changes in cellulose crystallinity, and crosslinking between aromatic units in lignin during superheated steam treatment [23]. The dimensional swelling rate declines because of the reduction in the hygroscopicity of cell walls. Moreover, the nutrients that microbes feed on are reduced by thermal decomposition, which endows bamboo with good resistance to microbes [24]. Hemicellulose, an important nutritive source of mildew, is significantly degraded by thermal treatment [25]. In addition to the decrease in hydrophilicity, the growth of fungi is inhibited in heat-treated bamboo. However, heat treatment has a negative influence on the mechanical properties of bamboo. It has been shown that the modulus of rupture (MOR) and modulus of elasticity (MOE) of bamboo decrease when bamboo is heated at 160 °C for over 10 min [26]. A more significant decrease is found as the temperature exceeds 200 °C [27]. There is a positive correlation between mechanical properties and density. Since the pyrolysis of chemical components lowers the density of bamboo [27,28], the mechanical performance is worsened. Nevertheless, the density is adjustable when bamboo is processed into a scrimber. Decreased density due to thermal treatment may have little effect on the mechanical properties of bamboo scrimber.

In the study, bamboo was treated with superheated steam and the treated bamboo was subsequently used for the preparation of outdoor bamboo scrimbers. The resistance of the scrimbers to the actions of force, water, decay and mildew was systematically investigated. Meanwhile, the aging resistance was evaluated via the comparison of mechanical performance and dimensional stability. The aim was to reveal the effect of heat treatment temperature on the durability of outdoor bamboo scrimbers, which may provide the heat treatment of bamboo scrimbers for outdoor use with a technological reference.

## 2. Materials and Methods

### 2.1. Materials

Four-year-old fresh Ci bamboo (*Neosinocalamus affinis*) with a diameter of 30–60 mm was collected from a natural forest in Sichuan Province, China. Phenolic resin with a solid content of 43%, a viscosity of 157 mPa·s at 25 °C, and a pH of ~13.3 was obtained from Dynea Chemical (Beijing, China) Co., Ltd. Deionized water was obtained from Millipore Milli-Q system. Other chemicals were of reagent grade and were purchased from Aldrich Chemical (Shanghai, China) Co., Ltd.

### 2.2. Heat Treatment of Bamboo Fibrous Mats

Bamboo was processed into fibrous mats according to the reported method [29]. The mats were treated with superheated steam at various temperatures. In brief, the mats were heated to 100 °C in the sealed box and then superheated steam was loaded. The inner temperature was slowly raised to the given temperature (i.e., 160 °C, 180 °C and 200 °C) and held for 2 h. After that, the mats were slowly cooled to 100 °C while the superheated steam was maintained until the inner temperature reached room temperature.

The chemical compositions of bamboo were quantitatively determined by the reported method [20], where the relative content of holocellulose, α-cellulose, and acid-insoluble lignin was measured according to China Standard GB/T 2677.10-1995, GB/T 744-1989 and GB/T 2677.8-1994, respectively. The content change rate (*CL*, %) of the three components after heat treatment was calculated by Equation (1).

(1) $$CL=\frac{c_{2}-c_{1}}{c_{1}}\times 100\%$$

where *c*_1_ and *c*_2_ were the relative content of the three components before and after the heat treatment, respectively.

Fourier transform infrared spectroscopy (FT-IR) was collected using a Nicolet Nexus 670 spectrometer (Thermo Scientific, Waltham, MA, USA) equipped with a Thermo Nicolet Smart Golden Gate MKII Single reflection ATR accessory. The sample slices with clean surfaces were placed on the diamond crystal and the collection was performed in the wavenumber range from 4000 to 400 cm^−1^ with a resolution of 4 cm^−1^ and 64 scans.

### 2.3. Bamboo Scrimber Fabrication

Scrimber samples were prepared according to the reported method [11]. The mats were impregnated with diluted phenolic resin and dried at 50 °C to a moisture content of approximately 8%. A certain weight of the impregnated mats was assembled along the grain in the mold. The hot pressing was conducted at 145 °C under the pressure of 4.0 MPa for 15 min to obtain the samples with a given density of 1.10 g/cm^3^. All samples were then conditioned in a room at 20 °C, 65 RH% for 2 weeks.

### 2.4. Mechanical Test

The properties of static bending and short-beam shearing were tested according to the standard GB/T 17657-2013 and GB/T 20241-2006, respectively. The sample dimension was 240 mm(L) × 20 mm(W) × 12 mm(T) for the bending test and 72 mm × 40 mm × 12 mm for the shearing test. All tests were performed on a CMT5105 universal testing machine (MTS systems, Shanghai, China). For each identical condition, at least seven duplicates were measured to ensure good reproducibility.

### 2.5. 28-Hour Heating Cycle Test

The tests were conducted according to China standard GB/T 17657-2013, in which the water absorption and thickness swelling rate were measured. In detail, scrimber specimens with a dimension of 50 × 50 × 12 mm^3^ were completely immersed in boiling water for 4 h, then dried in an oven at 60 °C for 20 h, and subsequently immersed in boiling water for another 4 h again. The water absorption (*WA*) and thickness swelling rate (*TS*) of bamboo scrimbers were calculated according to Equations (2) and (3), respectively.

(2) $$WA=\frac{w_{2}-w_{1}}{w_{1}}\times 100\%$$

(3) $$TS=\frac{t_{2}-t_{1}}{t_{1}}\times 100\%$$

where *w*_1_ and *w*_2_ were the specimen weight before and after testing, respectively; *t*_1_ and *t*_2_ were the specimen thicknesses before and after testing, respectively.

### 2.6. Accelerated Aging Test

Bamboo scrimbers were subjected to six complete cycles of accelerated aging according to GB/T 17657-2013: (1) Immersion in water at 49 ± 2 °C for 1 h, (2) exposure to steam at 93 ± 3 °C for 3 h, (3) freezing at −12 ± 3 °C for 20 h, (4) heating at 99 ± 2 °C in dry air for 3 h, (5) exposure again to steam at 93 ± 3 °C for 3 h, and (6) heating in dry air at 99 ± 2 °C for 18 h. After the completion of the six-cycle accelerated aging, the specimens were conditioned at 20 °C, 65 RH% for at least 48 h before use. Subsequently, the mechanical test in Section 2.4 and the determination of the thickness swelling rate were conducted.

### 2.7. Artificial Weathering Test

The weathering tests were carried out according to GB/T 16422.2-99(2000) standard. The tangential surfaces of bamboo scrimber were exposed under Xenon-arc light for 2 h which included 102 min for drying and 18 min for water spray. When 96 complete cycles of exposure were done, their mechanical properties were tested according to Section 2.4, and the thickness swelling rate was measured as well.

### 2.8. Decay Resistance Test

Decay resistance tests were performed according to GB/T 13942.1-2009 standard. The fungi included a white-rot fungus *Phanerochaete chrysosporium Burdsal* and a brown-rot fungus *Gloeophyllum trabeum (Pers.) Murrill*. Scrimber specimens with a size of 20 mm × 20 mm × 5 mm were dried at 103 ± 2 °C in the oven to a constant weight (*m*_1_), placed on the culture medium with mono fungus, and kept at 28 °C and 75 RH % in darkness for 12 weeks. Afterward, they were removed from the culture medium and the mycelium on the surface was scraped off. The cleaned specimens were oven-dried at 103 ± 2 °C to a constant weight and the weight (*m*_2_) was recorded. The mass loss rate (*ML*, %) was calculated by Equation (4).

(4) $$ML=\frac{m_{1}-m_{2}}{m_{1}}\times 100\%$$

The mass loss was used to determine the decay resistance grade, which is classified according to the specification as illustrated in Table 1.

### 2.9. Anti-Mildew Test

The tests were conducted on bamboo scrimber surfaces in Petri dishes according to GB/T 18261-2000. The fungi including *Trichoderma harzianum Rifai*, *Penicillium purpurogenum Stoll*, *Aspergillus fumigatus Fres* and *Botrydiplodia theobromae Pat.* Were provided by Research Institute of Wood Industry, Chinese Academy of Forestry. The specimen was cut into a rectangular sheet with 50 mm × 20 mm × 5 mm and placed on the glass rods where it was 3 cm far away from the medium. The infection degree was scored from 0 to 5 corresponding to the mildew coverings of 0%, 0–20%, 20–40%, 40–60% and 80–100%, respectively.

## 3. Results and Discussion

### 3.1. Chemical Analysis of Heat Treated Bamboo

#### 3.1.1. Chemical Composition

Bamboo is a type of biomass, which is susceptible to elevated temperature. After heat treatment, the relative content change rates of the main chemical compositions in bamboo are presented in Table 2. Heat treatment exerts a perceptible effect on the content of holocellulose, α-cellulose and lignin. After heat treatment, the contents of holocellulose and α-cellulose decreased while lignin content increased.

There was no substantial loss in holocellulose and α-cellulose after heat treatment at 160 °C. After heating at 180 °C, the content of the two components reduced by 2.48% and 9.04%, respectively. When heating at 200 °C, a significant reduction in the content of holocellulose and α-cellulose was observed, as shown in Table 2. Their relative contents reduced by 10.21% and 21.72%, respectively. This can be attributed to the degradation of cellulose and hemicellulose. Holocellulose is mainly composed of hemicellulose and cellulose. Hemicellulose has poor thermal stability and thus easy hydrolysis occurs during the heat treatment of bamboo. Cellulose possesses great thermochemical properties, but the degradation of hemicellulose gives rise to a decrease in the relative content of cellulose. When hemicellulose is heated over 140 °C the acetyl group in its chains gets hydrolyzed into acetic acid [30]. Acetic acid can catalyze the degradation of polysaccharides, including cellulose and hemicellulose [23,31]. Therefore, the relative contents of holocellulose and α-cellulose were decreased by the heat treatment. The increase in the relative content of lignin was ascribed to the decrease in holocellulose content.

#### 3.1.2. Chemical Structure

**Figure 1:**
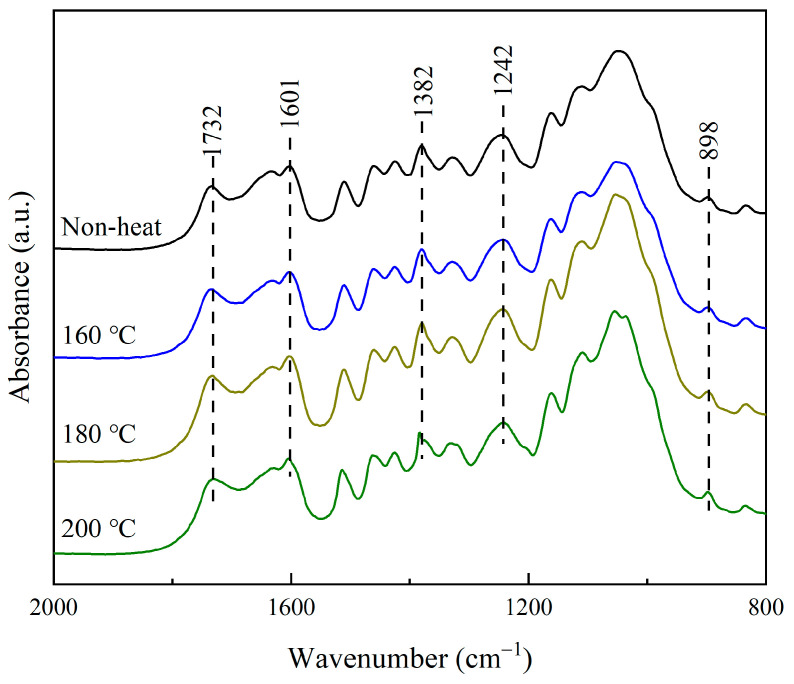
FT-IR spectra of the untreated and treated bamboo.

The chemical structure of bamboo after heat treatment was detected with an FT-IR spectrometer. Figure 1 shows the fingerprint area with a wavelength between 1800 to 800 cm^−1^, which can provide basic information about the changes in chemical composition.

**Figure 2:**
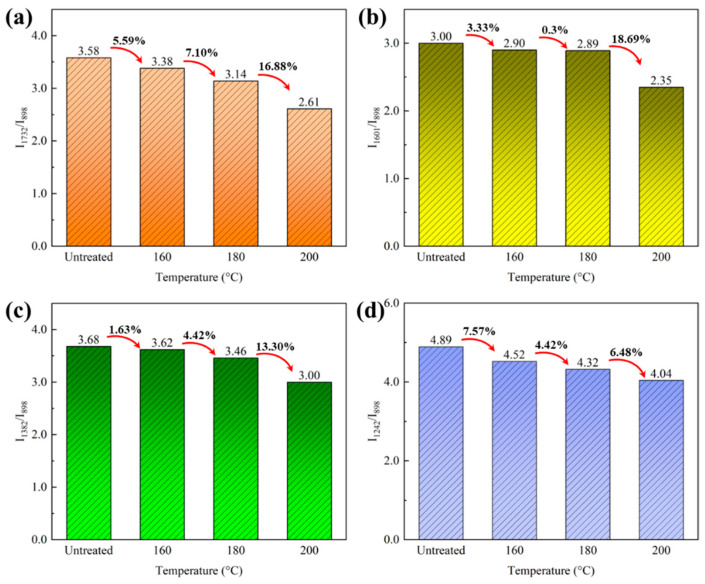
Relative intensity of characteristic peaks of the untreated and treated bamboo: (**a**) *I*_1732/898_, (**b**) *I*_1601/898_, (**c**) *I*_1382/898_, and (**d**) *I*_1242/898_.

The characteristic peak at 898 cm^−1^ is assigned to C-H deformation in cellulose. It has been reported that the peak was assumed to be essentially unaltered at elevated temperatures [32,33]. Thus, the peak was chosen as a reference peak for spectrum normalization. The relative intensity of the representative peaks was calculated based on the intensity of the reference peak, and the results are shown in Figure 2.

The peak at 1730 cm^−1^ is assigned to the stretching vibration of a non-conjugated carbonyl group in xylan. Its relative intensity decreased in the treated bamboo, which demonstrated the hemicellulose degradation during the heat treatment of bamboo. It was confirmed by the decrease in peak intensity at 1382 cm^−1^ assigned to C-H bending vibration in cellulose and hemicellulose. The results were consistent with the observation of the chemical composition analysis in Section 3.1.1. The absorption intensity of the peak at 1601 cm^−1^ assigned to the C=C aromatic skeletal vibrations of a benzene ring also declined after heat treatment. The benzene ring is a specific structure of lignin in bamboo, and the decrease in its related peak intensity suggested a change in the chemical structure of lignin during heat treatment. In addition, the peak intensity at 1242 cm^−1^ assigned to the syringyl ring and C-O stretching vibration in lignin and xylan decreases in intensity. Therefore, the superheated steam treatment led to the degradation of cellulose and hemicellulose and the chemical change of lignin.

### 3.2. Mechanical Properties

#### 3.2.1. Bamboo Scrimbers with Superheated Steam Treatment

**Figure 3:**
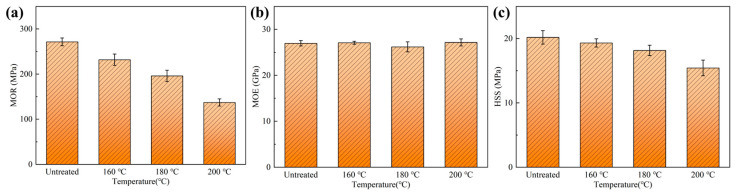
Mechanical properties of bamboo scrimbers after heating at various temperatures: (**a**) MOR, (**b**) MOE and (**c**) HSS.

The flexural properties of the heat-treated bamboo scrimbers are presented in Figure 3. Bamboo scrimbers have excellent performance due to the high strength of bamboo fibers [34]. The scrimbers with a density of 1.10 g/cm^3^ had a modulus of rupture (MOR) of approximately 270 MPa. But the strength dropped after heat treatment. It was attributed to the degradation of chemical compositions such as cellulose, which decreased the stiffness of bamboo cell walls. Previous research has reported that there was a highly positive correlation between strength and cellulose content [35,36]. The lowering content of cellulose gave rise to a decrease in MOR. It has been found in Section 3.1 that the higher temperature resulted in bigger cellulose loss. Therefore, higher temperatures led to lower bending strength of bamboo scrimbers as well. As the temperature elevated to 200 °C, MOR dropped to 137 MPa, which was only half of that of the untreated scrimbers. Similarly, MOE decreased. However, the bending properties still meet the requirements of structural bamboo scrimber specified in Chinese standard LY/T 3194-2020.

The short-beam shearing test can evaluate the bonding quality of laminated panels according to the theorem of conjugate shearing stress [37]. The horizontal shearing strength (HSS) of the untreated and treated bamboo scrimbers is shown in Figure 3c. Strength reduced after bamboo was treated with superheated steam, indicating that heat treatment affected the adhesive bonding performance of bamboo fibers. Studies demonstrated that many extractives during heat treatment emerged on the bamboo surface, leading to low surface wettability [25]. Since the surface could not be adequately accessed by adhesives, there was an adverse impact on the formation of a strong adhesive interface between bamboo fibers and adhesives, resulting in poor bonding properties. In addition, the pyrolysis of chemical components, particularly hemicellulose, has a great influence on the bonding [38]. Pyrolysis products could retardant the curing reaction of adhesives, thus adversely affecting the adhesive property.

#### 3.2.2. Bamboo Scrimbers after Accelerated Aging Test

Bamboo scrimbers with heat treatment display worsened mechanical properties. As bamboo scrimbers underwent the accelerated aging test, those properties became worse due to stress failure stemming from the dry shrinkage and wet expansion of cell walls. To study the influence of the heat treatment temperature on the aging resistance of bamboo scrimbers, the loss rate (*LR*) of MOR, MOE and HSS were calculated with Equation (5):

(5) $$LR=\frac{S_{f}-S_{b}}{S_{b}}\times 100\%$$

where *S_b_* and *S_f_* were the strength of bamboo scrimber before and after the accelerated aging tests, respectively.

**Figure 4:**
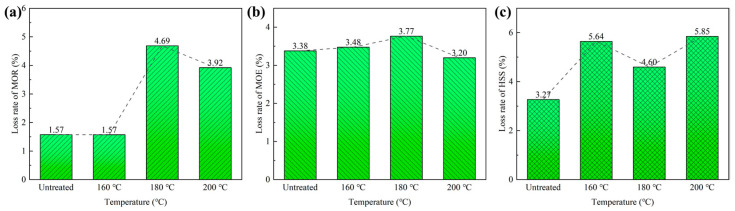
Loss rates of (**a**) MOR, (**b**) MOE and (**c**) HSS of bamboo scrimbers after the accelerated aging test.

The results are visually presented in Figure 4. MOR of the untreated scrimbers decreased by 1.57% after the accelerated aging test. Heat treatment of bamboo at 160 °C did not change the MOR loss rate after the accelerated aging test, suggesting that heat treatment at 160 °C had little influence on the bending strength. However, the loss rate was 4.69% at 180 °C, which was approximately triple that of the untreated scrimbers. When the temperature was raised to 200 °C, the loss rate was much higher than that of the untreated ones. Those indicated that the treatment temperature above 160 °C had a negative influence on the bending strength of bamboo scrimbers. The loss rates of MOE for all bamboo scrimbers ranged from 3.20% to 3.80%, suggesting that heating temperature had little influence on the elastic modulus of bamboo scrimber.

The accelerated aging tests reduced the HSS of bamboo scrimbers without heat treatment by 3.27%. As for the scrimbers with heat treatment, HSS decreased by over 4.50%, which indicated that the heating treatment temperature exerted a negative effect on the shearing strength. Therefore, the superheated steam treatment did not improve the mechanical properties of bamboo scrimbers in accelerated aging conditions.

#### 3.2.3. Bamboo Scrimbers after the Artificial Weathering Test

**Figure 5:**
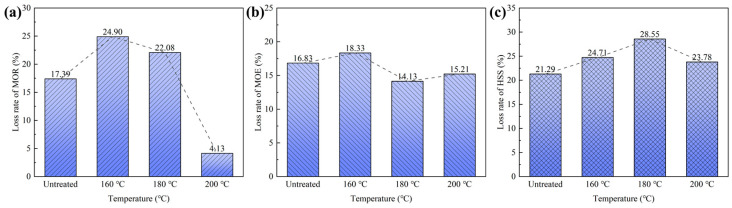
Loss rates of (**a**) MOR, (**b**) MOE and (**c**) HSS of bamboo scrimbers after the artificial weathering test.

Bamboo scrimbers in the open, exposed to sunshine and water. The artificial weathering tests were employed to investigate the resistance of bamboo scrimbers to natural weathering. The loss rates of the mechanical index are shown in Figure 5. After 96 complete cycles of exposure, bamboo scrimbers had decreased mechanical properties. The scrimbers without heat treatment had a loss rate of 17.39% in MOR. As bamboo was treated at 160 °C, the loss rate reached 24.90%. However, the loss rate started to shrink when the temperature exceeded 160 °C. When the temperature was 200 °C, the loss rate was 4.13%, which was much lower than that of the untreated scrimbers. In addition, Figure 5b showed that the temperature above 180 °C led to a low loss rate of MOE. Those suggested that high temperatures can improve the bending properties of bamboo scrimbers to resist weathering attacks.

Figure 5c shows the loss rates of the short-beam shearing strength of bamboo scrimbers. For the untreated scrimbers, HSS decreased by 21.29% after the weathering test. When the heating temperature elevated to 180 °C, the loss rate increased and the loss rate reached its maximum level (i.e., 28.55%). At the heating temperature of 200 °C, the loss rate lessened to 23.78%, which was higher than that of the scrimbers without heat treatment. Accordingly, it can be concluded that superheated steam treatment could not improve the mechanical properties of bamboo scrimbers in artificial weathering conditions.

### 3.3. Water Resistance

#### 3.3.1. 28-Hour Heat-Moisture Cycle Test

**Figure 6:**
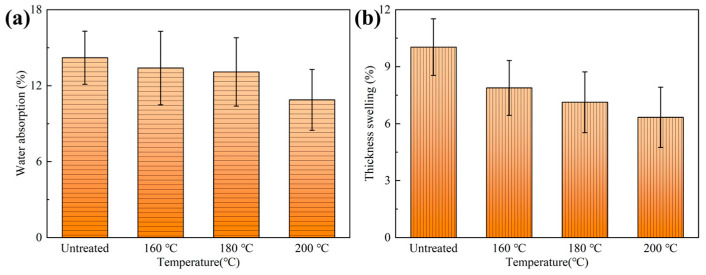
(**a**) Water absorption and (**b**) thickness swelling of scrimbers made from heat treated bamboo.

The water absorption rates of bamboo scrimbers after the 28-h heating cycle test are depicted in Figure 6. The untreated bamboo scrimbers had a water absorption rate of approximately 14%. After bamboo was treated by superheated steam, the water absorption became low. At the temperature of 160 °C, the water absorption rate was 13.40% on average, which decreased by 5.7% compared to the untreated scrimbers. When the temperature was 200 °C, the water absorption rate decreased to 10.87% which was 23.50% lower than that of the untreated bamboo scrimbers. Those results were closely related to the change in bamboo chemical compositions. There are a lot of hydrophilic groups such as the hydroxy group, and carboxyl group in bamboo cell walls, which play a main role in the hygroscopicity of bamboo scrimbers [39]. The heat treatment reduced their content in bamboo, thus leading to low hygroscopicity.

The absorbed water caused the thickness swelling of cell walls, which was macroscopically illustrated by the increase in the thickness of bamboo scrimbers. As presented in Figure 6b, the thickness swelling rate of the control scrimbers was 10%. After heat treatment was performed on the bamboo at 160 °C, 180 °C and 200 °C, the thickness swelling of the resultant scrimber declined by 21%, 29% and 37%, respectively, suggesting that high temperature contributes to a smaller thickness swelling rate. Therefore, the superheated steam treatment of bamboo can remarkably enhance the water resistance of the resultant bamboo scrimbers.

#### 3.3.2. Accelerated Aging Test

**Figure 7:**
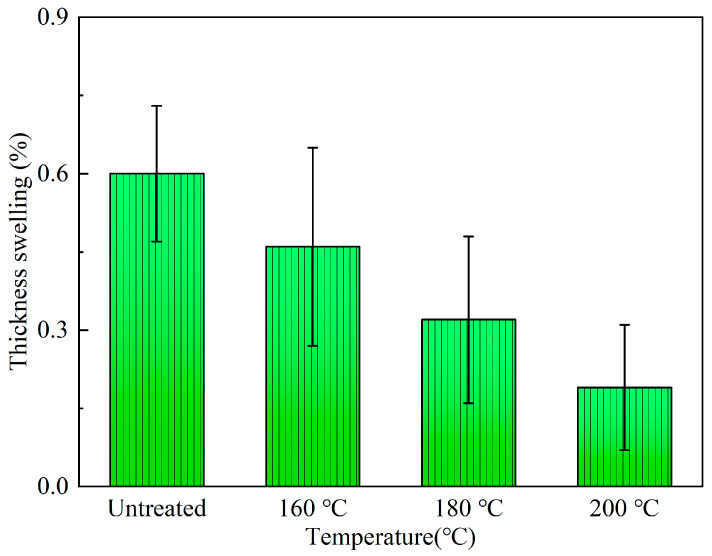
Thickness swelling of bamboo scrimbers after the accelerated aging test.

The thickness swelling rates of bamboo scrimbers after the accelerated aging tests are shown in Figure 7. The thickness swelling rate of the scrimbers made from the untreated bamboo was only 0.60%, As bamboo was treated at 160 °C, thickness swelling decreased to 0.46%. Besides, the thickness swelling exhibited a decreasing trend with the elevation of temperature. When the temperature was 200 °C, the thickness swelling was as low as 0.19%. Those suggested that the heat treatment can improve the dimensional stability of bamboo scrimbers during accelerated aging and the dimensional stability can be further enhanced by raising the temperature for treating bamboo.

#### 3.3.3. Artificial Weathering Test

**Figure 8:**
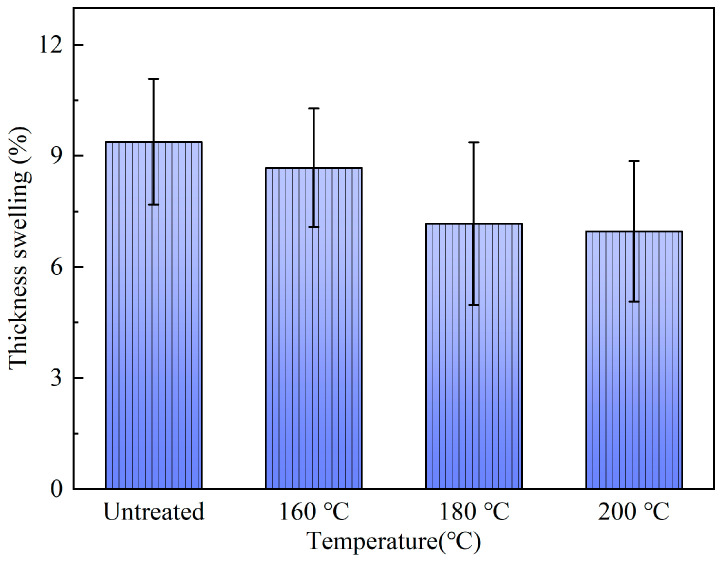
Thickness swelling of bamboo scrimbers after the artificial weathering test.

Figure 8 shows the thickness swelling rates of bamboo scrimbers after irradiation with a xenon lamp and spraying with water. The thickness swelling rate was 9.38% for the control scrimbers. When bamboo was treated with superheated steam, the rate declined. The thickness swelling rate at 160 °C was 8.68% and it decreased to 6.96% at 200 °C. Those indicated that the thickness swelling rate decreased as the temperature was raised. It thus can be concluded that heat treatment can effectively improve the weathering resistance of bamboo scrimbers.

### 3.4. Decay Resistance

**Figure 9:**
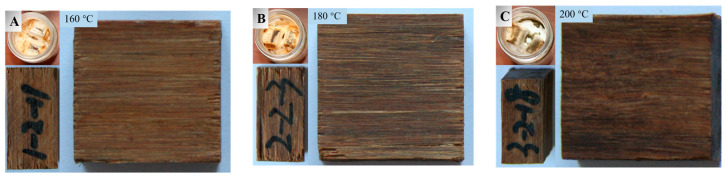
Surfaces of (**A**) 160, (**B**) 180 and (**C**) 200 °C -treated bamboo scrimbers attacked by white-rot fungi.

**Figure 10:**
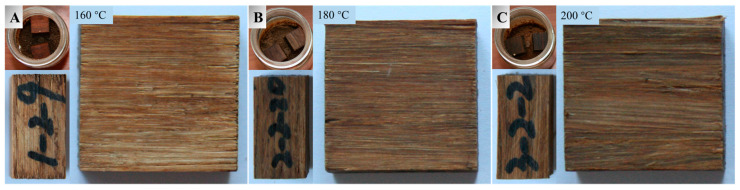
Surfaces of (**A**) 160, (**B**) 180 and (**C**) 200 °C -treated bamboo scrimbers attacked by brown-rot fungi.

There are a lot of fungal spores in the natural world that are too small to be directly observed with the naked eye. They corrode the lignocellulosic materials all the time. Since bamboo contains adequate nutrients such as glycoprotein and pectin, it is vulnerable to fungi attacks. This is one main obstacle for diversifying the applications of bamboo-based materials. Herein, bamboo scrimbers made from heat treated bamboo were infected separately by a white-rot fungus (*P. chrysosporium*) and brown-rot fungus (*G. trabeum*) to study their resistance to rot fungi. It can be seen from the culture bottles in Figure 9 and Figure 10 that the surfaces covered by fungi exceed 90% for all bamboo scrimbers. After 4 weeks, obvious marks were observed on the surfaces of the bamboo scrimbers, suggesting that the humid environment, nutrients, and oxygen on the scrimbers were appropriate for fungal reproduction. The mass loss rates of bamboo scrimbers after the fungi attacks are shown in Table 3.

It can be seen in the table that the mass loss of all bamboo scrimbers was less than 6%, which met the requirements for Grade I (*ML* ≤ 10%) as specified by China standard GB/T 13942.1-2009. This indicated that bamboo scrimbers had strong corrosion resistance. With the elevated temperature, the mass loss rate was reduced. As the temperature was 200 °C, the mass loss by white rot and brown rot was 3.42% and 2.71%, respectively. They were 32% and 39% lower than that of the control scrimbers. Low weight loss meant better decay resistance. With the increase in temperature, more nutrients that fungi feed on are degraded, resulting in improved decay resistance. Therefore, heat treatment of bamboo can markedly improve the decay resistance of resulted scrimbers.

### 3.5. Anti-Mildew Properties

**Figure 11:**
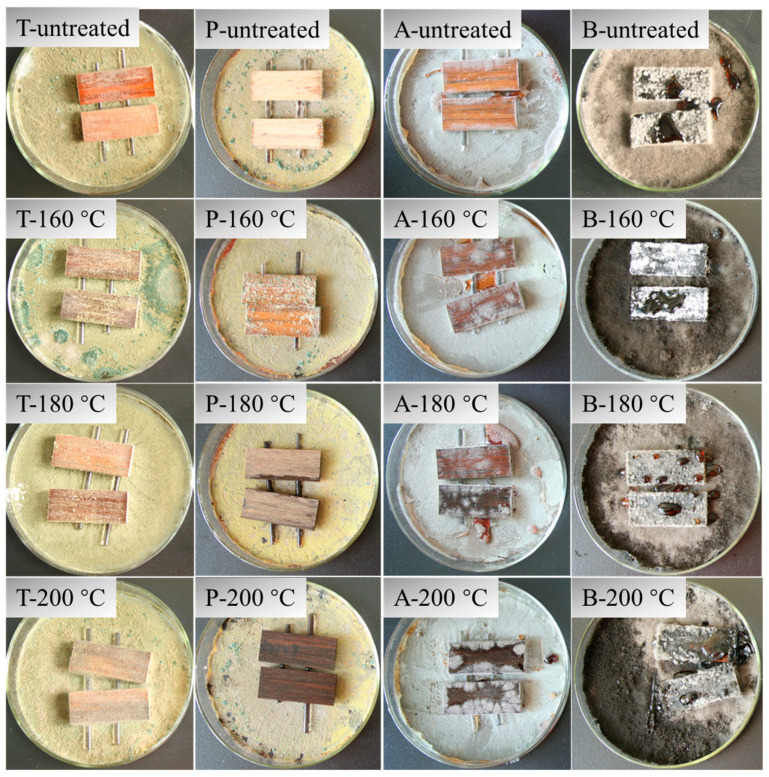
Surfaces of bamboo scrimbers stained by (T) *T. harzianum*, (P) *P. purpurogenum*, (A) *A. fumigatus* and (B) *B. theobromae*.

Bamboo scrimbers retain the natural stripes of bamboo, which thus are loved by the public. Figure 11 shows the surfaces of bamboo scrimbers stained by four common species of mildew. It was found that the surfaces of bamboo scrimbers were covered by mildew to different extents, which reflected that bamboo scrimbers have different levels of anti-mildew properties. The infection values were used to evaluate the anti-mildew levels and are presented in Table 4.

The infection values by *Trichoderma harzianum* and *Botrydiplodia theobromae* were 4 for the control bamboo scrimbers. As bamboo was with superheated steam, the values of 4 remained on the resultant scrimbers, suggesting that heat treatment doesn’t hold out against the attack of the two mildews. However, bamboo scrimbers with heat treatment had a certain resistance to the two mildews *Penicillium purpurogenum* and *Aspergillus fumigatus*. The infection values of the untreated scrimbers were 3.8 and 4 for *P. purpurogenum* and *A. fumigatus*, respectively. When bamboo scrimbers were involved with thermal treatment, the values decreased. The scrimbers treated at the temperature of 200 °C, for example, had infection values of 3.5 for *P. purpurogenum*, which is approximately 8% lower than that of the untreated ones. It indicated that heat treatment was marginally beneficial to improve the anti-mildew properties of bamboo scrimbers. Mildew reproduction depends on appropriate conditions such as temperature, humidity, oxygen and nutrients. Chemical components, especially the sugar and starch with high content, meet the requirements for mold growth [40]. Heat treatment reduces the content of starch and hemicellulose in bamboo, which effectively restrains mold growth in subsequent bamboo scrimbers. Consequently, the anti-mildew property of the bamboo scrimbers was improved via heat treatment. However, the anti-mildew effect was still poor, as demonstrated by high infection values. Therefore, other methods, such as coatings and antiseptics, are employed to further improve the anti-mildew properties of outdoor bamboo scrimbers.

## 4. Conclusions

In this study, bamboo was treated with superheated steam first. It was then used as the raw material for preparing bamboo scrimbers. The relative contents of holocellulose and α-cellulose but lignin in bamboo decreased after heat treatment. The mechanical properties, including bending and shearing strength of the bamboo scrimbers, slightly decreased when bamboo was heat treated, due to the degradation of main chemical compositions. The resistance to aging and weathering was enhanced by presenting excellent dimensional stability. The decay resistance reached Grade I and it can be dramatically enhanced by elevating the temperature for bamboo treatment. The anti-mildew properties were also improved, while scrimber interiors were not attacked by mildew. Taken together, superheated steam treatment of bamboo improved water resistance, decay resistance and anti-mildew properties of the resulted scrimbers by changing the chemical composition of bamboo, even though some mechanical properties were compromised.

## Figures and Tables

**Table 1:** Corresponding relationship between the mass loss and decay resistance grade.

ML (%)	Decay Resistance Grade
0–10	I (Strong decay resistance)
11–24	II (Decay resistance)
25–44	III (Slightly resistant to decay)
>45	IV (Not resistant to decay)

**Table 2:** Relative content change rates of bamboo chemical compositions after heat treatment.

Temperature (°C)	Chemical Composition Change (%)		
	Holocellulose	α-Cellulose	Lignin
160 °C	−1.39	−2.73	6.77
180 °C	−2.48	−9.04	11.51
200 °C	−10.21	−21.72	16.76

**Table 3:** Mass loss and decay resistance grade of bamboo scrimbers after fungi attacks.

Temperature (°C)	White-Rot Fungi		Brown-Rot Fungi	
	*ML* (%)	Decay Resistance Grade	*ML* (%)	Decay Resistance Grade
Untreated	5.06 (8.52) ^1^	I	4.46 (14.91)	I
160	4.34 (6.82)	I	3.72 (7.67)	I
180	4.12 (9.51)	I	3.37 (10.91)	I
200	3.42 (5.69)	I	2.71 (7.82)	I

^1^ The coefficient of variance (%) is given in parentheses.

**Table 4:** Infection value of bamboo scrimbers by four species of mildews.

Temperature (°C)	*T. Harzianum*	*P. Purpurogenum*	*A. Fumigatus*	*B. Theobromae*
Untreated	4	3.8	4	4
160	4	3.6	4	4
180	4	3.6	3.8	4
200	4	3.5	3.8	4

## Data Availability

The data used that support the findings of this study are available from the corresponding author (chinayuwj@126.com) upon reasonable request.
